# STEPS: An Indoor Navigation Framework for Mobile Devices

**DOI:** 10.3390/s20143929

**Published:** 2020-07-15

**Authors:** Yael Landau, Boaz Ben-Moshe

**Affiliations:** Department of Computer Science, Ariel University, Ariel 40700, Israel; yaelandau22@gmail.com

**Keywords:** sensor fusion, indoor localization, particle filter, Android indoor position

## Abstract

This paper presents a vision-based navigation system designed for indoor localization. The suggested framework works as a standalone 3D positioning system by fusing a sophisticated optical-flow pedometry with map constrains using an advanced particle filter. The presented method requires no personal calibration and works on standard smartphones with relatively low energy consumption. Field experiments on Android smartphones show that the expected 3D error is about 1–2 m in most real-life scenarios.

## 1. Introduction

Indoor positioning is an important capability for a widerange of applications, including location-based services (LBS), public safety (first responders), and autonomous robotics (indoor navigation). Whereas LBS-related applications mainly target smartphone users navigating in shopping malls [1,2], first responders might use foot-mounted pedometers (see [3,4,5]). Although several research groups presented indoor positioning systems (IPS) solutions in the last two decades, the robustness and accuracy of existing IPS often are insufficient [6]. Utilizing a particle filter for localization problems is common for both indoor and outdoor scenarios [7]. In essence, the **sense**, **action**, and **resample** functions of each implementation differs from one algorithm to another. Moreover, harnessing the smartphone’s internal sensors can be performed with a wide range of techniques. Although many types of applications require indoor pedestrian positioning, it seems the following properties should be optimized with respect to almost any such method:

**Accuracy**: Often the main and foremost parameter being tested
**Keep it simple**: Simplicity is a key factor: The system should work automatically with no manual overhead operation or calibration.
**Real time**: For natural and intuitive positioning results, especially for highly dynamic movements’
**Privacy**: The suggested solution should be able to work offline, e.g., “flight-mode”.
**Bring your own device**: The suggested solution should work on existing commercial off-the-shelf mobile devices (e.g., common smartphones). This requirement implies energy-consumption and computing-power limitations.

### 1.1. Related Works

In this section, we present a general overview of the research related to indoor navigation. In the last two decades, a massive amount of research has been conducted in the field of indoor positioning and navigation. However, unlike the case of global navigation satellite system (GNSS)—the outdoor navigation used in almost all smartphones—indoor navigation is somewhat more challenging because its expected accuracy is often insufficient. Further, as of 2020, no major indoor-positioning method with global positioning system-like performance existed. Moreover, many indoor positioning methods require complicated settings or mapping; others often are not robust enough for complicated, real-life indoor scenarios. For a recent survey on general indoor positioning methods see [8,9,10]. Below is a list of Indoor positioning methods, presented according to their underlying technology:

- **Indoor GNSS:** In general, GNSS signals are mostly unavailable in indoor scenarios but, in many cases, can be received through windows, skylights, or simply “thin” ceilings. Because it is poplar and widely used in smartphones, several researchers have suggested GNSS indoor positioning solutions [11].
- **WiFi Fingerprinting:** This method uses the signals of nearby wireless access points to determine its location relative to them [12].
- **3G/4G Cellular Indoor Positioning:** As in the case of WiFi, LTE, GSM or CDMA signals (4G and 3G cellular networks) can be mapped as RF fingerprinting. Notably, such a process can be performed on the client side or the base-station side. See, [13,14,15].
- **Bluetooth Low Energy (BLE):** This is another popular RF fingerprinting method [16].
- **Pedometer and Inertial Navigation:** This method computes a relative position with respect to a known starting location. It approximates both speed and direction via inertial motion sensors. Such an inertial system suffers from drifting error that accumulates with time, for example, [17].
- **Optical Flow:** This method calculates the user’s relative motion according to optical features. Comparing the features from consecutive frames allows a tight estimation of the relative motion. As with the pedometer method, the optical flow method tends to drift in time [18].
- **Magnetic Field:** As in WiFi and cellular signals cases, the magnetic field sensor can be used to generate unique magnetic “fingerprint” to certain locations. The magnetic filed is affect by electric devices and metal materials which distort the earth magnetic filed. The use of magnetic filed for indoor positioning was suggested by several researchers see: [19,20,21].
- **Visual Navigation:** This navigation method searches for some preknown landmarks in the field of view to locate itself respectively [22,23]. In general, this method can be addressed as SLAM: Simultaneous Localization And Mapping [24].

These methods can be divided roughly into three main categories: (a) RF fingerprinting (radiant sources), (b) step counter, or (c) optical flow. The first category, RF fingerprinting, requires existing (mapped) infrastructure. This is its main disadvantage, whereas the main fault of the other methods is their drifting nature. Our suggested system uses a mixed approach based on an advanced particle filter to achieve the best of all worlds.

### 1.2. Our Contribution

**Figure 1:**
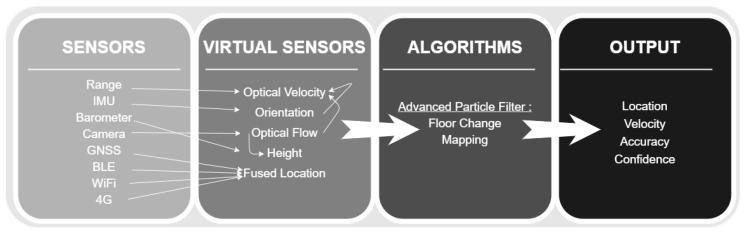
The general structure of the STEPS indoor positioning system (IPS) is composed of four layers: sensors (physical, smartphone based); virtual sensors, which can be thought of as dedicated-purpose conceptual sensors based on sensor fusion; algorithms (positioning, state estimation, mapping); and output, the last layer, required for continuance positioning (1–30 Hz), as well as for accuracy, confidence estimation, and event-based reporting, such as floor change.

The field of indoor positioning has been studied extensively. Yet, having a reliable indoor positioning sensor is still a significant challenge. In this article, we suggest a practical indoor positioning solution that is robust and relatively accurate. This work presents a smartphone IPS based on augmented reality (AR) and mixed reality tools, such as Google’s ARCore or Apple’s ARKit. The AR tools are used mainly as visual pedometry (scaled optical flow) sensors, which then are fused with an advanced version of the localization particle filter to produce a solution that is both accurate and robust for various indoor-positioning applications. Moreover, the presented method (named STEPS) allows a simple and efficient mapping solution. Combined with the localization particle filter, STEPS allows 1 m to 2 m positioning accuracy in most standard indoor scenarios (see Figure 1 for the general framework of STEPS).

### 1.3. Smartphone’s Sensors for Indoor Positioning

In this subsection we elaborate on the various sensors which are common in smartphones and can play a roll in indoor positioning. Table 1 presents the main sensors which are common in smartphones and their main usage related to indoor positioning.

## 2. Particle Filter for Localization

### 2.1. Basis of Indoor Positioning

The user global position can be retrieved from existing geolocation services (e.g., Google Maps geolocation API). The user location commonly is approximated using RF signals (4G/3G, wireless local area network, BLE, and even weak GNSS). The accuracy of such methods is considered to be at the “building” (10 m to 30 m) or “room” (5 m to 10 m) levels.

The user’s relative position often is computed using a pedometer. Smartphone-based pedometers are composed of two major virtual sensors: (a) a “step counter”, which detects discrete step events and (b) an orientation sensor, which approximates the user’s global/relative direction. Combined, the two parts allow step-based relative path computations. Naturally, such a method tends to drift in time (and steps). To tackle the drifting and the measurement-noise issues, we applied a filtering algorithm.

### 2.2. Particle Filter for Localization

This section discusses a possible naive particle filter algorithm for localization estimation. Because the particle filter method represents the posterior distribution of a set of particles *P* (|P|=n) on a given map, the result of this algorithm is, for each step, a new set of particles $P^{\prime }$ with a (slightly) different distribution. The goal of this algorithm is to converge all the particles into one area on the map in a few steps. After converging, the internal state (location) will be the average location of the best particles (i.e., those with the highest grades). Before presenting the algorithm, some terms must be clarified:

- **Map:** The particle filter method estimates the internal state in a given area. Thus, the input of this algorithm is a 3D map of the region of interest (ROI). This map should include as many constraints as possible (e.g., walls and tables). The map constraints are one of the parameters that determine each particle grade, because particles with impossible locations on the map will be downgraded.
- **Particle:** At the beginning of the localization process, we “spread” a set of particles *P* on the map. Each particle $x_{i}\in P$ will have the attributes of location: <x, y, z>, orientation: *w*, and grade: *g*. In each step, all particles’ locations, orientations, and grades will be modified. Because these particles represent the internal-state distribution, the sum of the *P* particles’ grade is 1 in each step. At the initial step, each particle $x_{i}$ grade is $\frac{1}{\left|P\right|}$. The particle’s grade will be set higher as its location on the map seems more likely to represent the internal state.
- **Move (action) function:** With each step, all particles on the map should be relocated according to the internal movement. Hence, for each step, we calculate the movement vector (in 3D) and the difference in orientation, then move all particles accordingly. As commonly used with smartphones, the mobile pedometer (step counter with orientation) provides the movement in each step.
- **Sense function:** The device sensors also are used to determine each particle grade. The sense method predicts each particle’s sense for each step and then grades it with respect to the correlation between the particle prediction and the internal sense. In our case, the sense function is discrete, comparing each particle’s internal location to the corresponding location on the map. The grading is according to the match between the map location and a known state (going up or down, inability to walk thru walls, etc.).
- **Resampling:** The process of choosing a new set of particles $P^{\prime }$ ($|P^{\prime }|=n$) from *P* can be conducted with various methods, but the main purpose of resampling is to choose the particles with high grades over those with low grades.
- **Random noise**: To prevent convergence of the particles from happening too quickly (and thus risk missing the true location), after resampling, we move each particle with a small random noise on the map. Usually, this is done by moving each particle in a small radius from its original location.

**Figure 2:**
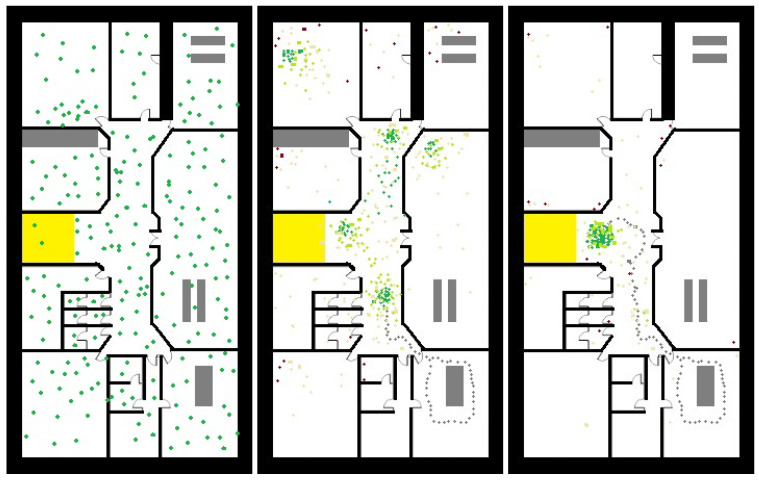
The basic particle filter process. (**Left**): particles are uniformly spread within the region of interest (ROI). (**Center**): after a short walk (about 12 m), the particles begin to cluster around locations with high likelihood. (**Right**): after a total walk of about 25 m, the particles converge to a single position cluster. Note: Some particles still are far from the main cluster, but the expected error (which corresponds to the area) is relatively low.

Algorithm 1 and Figure 2 explain the particle filter method process using mobile pedometry sense.

**Algorithm 1:** Generic particle filter localization algorithm: A black-and-white map is used to present the geo-constrains used by the particle filter.

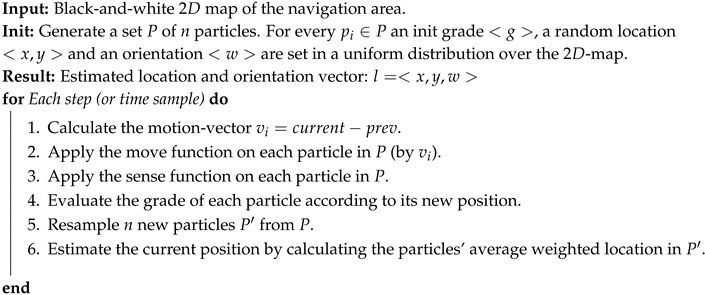



### 2.3. Run-Time Analysis

This subsection analyzes the theoretical runtime of a particle filter for localization. In general, the runtime of the particle filter is proportional to the number of particles (linearly). Consider Algorithm 1: lines 2–6 perform an O(1) operation on each particle and line 1 is performed on a sensor level and can be seen as a constant time operation (independent of the input size). The analytical runtime of resampling (line 5) is somewhat implementation dependent yet, there are linear time algorithms for resampling see [25]. Thus, reducing the computation complexity of the algorithm can be done mainly by the following three approaches:

- Reducing the number of sample per s: such method can be done by using an adaptive sampling rate, i.e., in case of stationary user (low movement) a sampling rate of a 1 Hz should sufficient while in high dynamic movement (e.g., a running user) a sample rate of 30 Hz might be needed.
- Reducing the number of particles: such natural approach can be implemented using the expected probabilistic space represented the particle. Additional details regarding such improvement can be found in the improved algorithm see Section 3.7.
- Particle filter algorithms can be implemented efficiently on multi cores and GPGPU (General Purpose Graphic Processing Unit) platforms: [26]. Consider Algorithm 1, lines 2–4 can be perform in parallel (thread-core for each particle). Resampling (line 5) in calculating the “best location” (line 6) in parallel is somewhat tricky—yet several papers have suggested parallel and distributed solutions for these methods (e.g., [27,28]).

Using the above performance improvements we expect that the runtime and energy consumption of particle filters algorithms can be improved significantly, by running it on GPGPU enabled devices such as modern smartphones and NVIDIA’s Jetson boards.

## 3. Improved Algorithm

The naive algorithm is relatively time efficient. However, its precision might be insufficient in cases of large areas with few constraints. In this section, we propose a particle-filter-based algorithm with advanced methods to improve the results’ accuracy and robustness.

### 3.1. Map Constrains

Most particle filter algorithms use map constraints for evaluation. Traditionally, the map held two type of constraints—accessible and inaccessible areas. Our advanced particle filter algorithm also relies on the existence of a pre-made map of ROI, but with more constraints. Assembling such a map is an issue in itself. Such map is assembled by our system, using to the following technique:

- AR measurement tools for surface detection, which allowed us to determine the sampled ROI boundaries, and
- the map in the form of a painted image, using defined colors (A, B, C, D) to represent the verity of the constraints.

The colors are placed on the map according to the following logic:

- **A**: accessible area
- **B**: inaccessible area, such as walls or fixed barriers, as sensed by the AR tool
- **C**: partially accessible regions, representing locations with relatively low probability for users to be at (e.g., tables)
- **D**: floor-changing regions, such as stairs, escalators, and elevators

**Figure 3:**
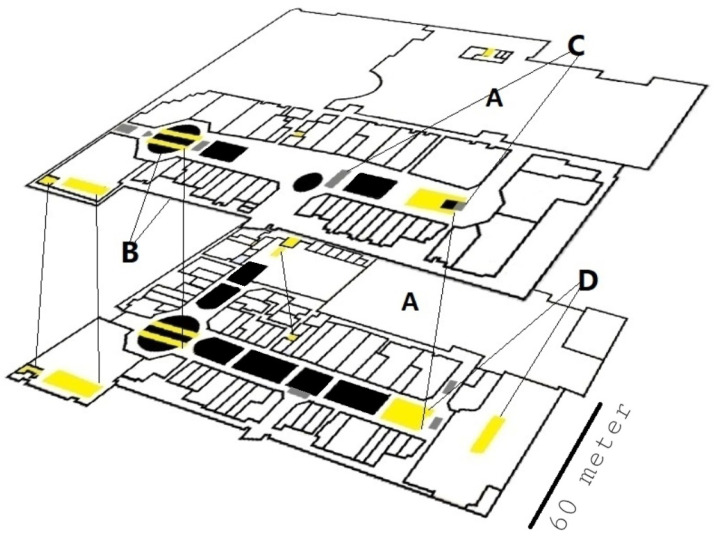
2.5D multicolored map example used in the advanced algorithm. **White** (marked by the letter “**A**”) represents accessible areas; **black** (marked as “**B**”) represents restricted regions (in this case, walls); **gray** (“**C**”) represents dynamic inaccessible areas (tables, in this case); and **yellow** ( “**D**”) represents stairs and elevators, in which elevation change is possible.

**Figure 4:**
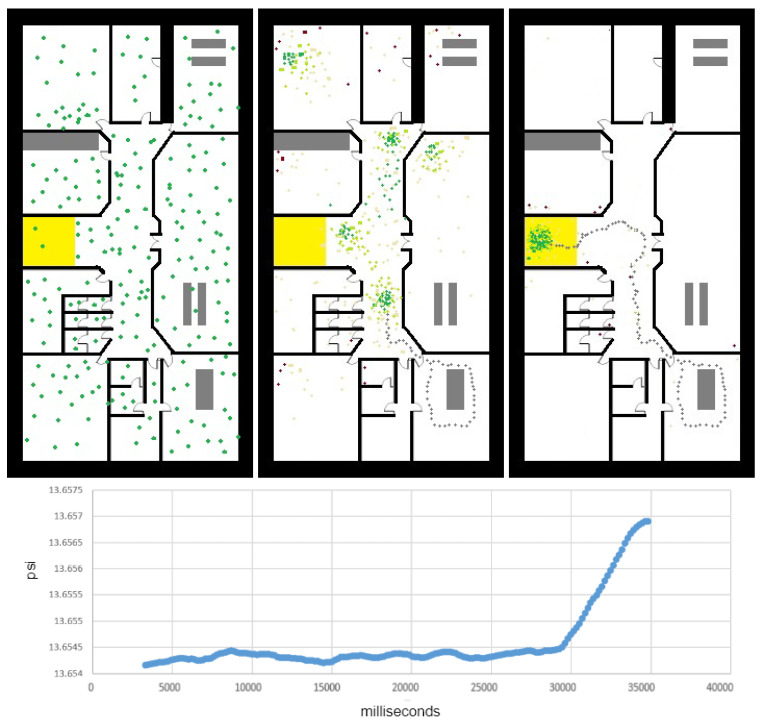
Particle filter for localization. (**Left**): Init state, the particles are uniformly distributed. (**Center**): using the short motion vector, the particles are beginning to organize into a few clusters. (**Right**): the particles converge to a single position cluster, mainly due to floor-change detection. The lower graph shows the barometer raw measurements (PSI) in time. The peak in the PSI measurements corresponds to the 3 m change between the two floors. The detection of floor change allowed the algorithm to converge efficiently to the right 3D location.

A 2.5D map, such as presented in Figure 3 and Figure 4 will be the base for the particle filter algorithm and later will be used to determine particle grades.

### 3.2. Floor-Change Detection

**Figure 5:**
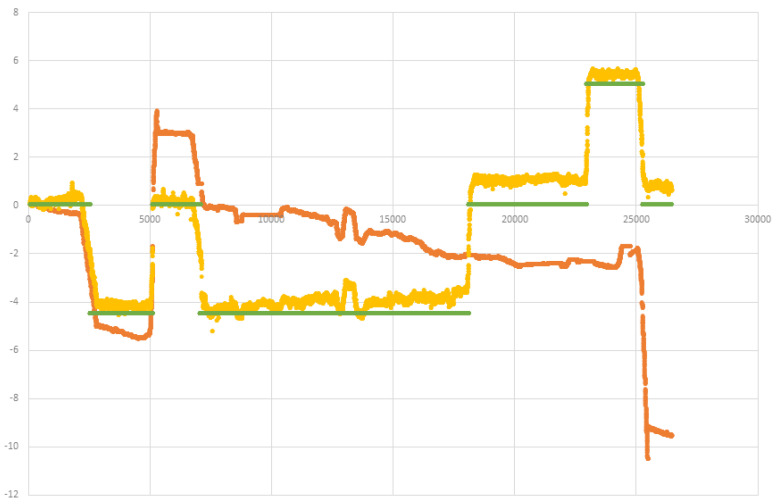
Height in meter over time, as measured by optical-flow sensor (orange), barometer sensor (yellow), and ground truth (green), demonstrate the superiority of the barometer-sensing method: The barometer’s accuracy was more than 1 m, whereas (due to varying light conditions) the optical flow performed poorly.

To generalize the localization algorithm from 2D to 3D (i.e., 2.5D), we should define a method to detect floor change. A “wrong floor” error is significant for the user and may cause critical errors related to wrong constrains applied by the “wrong map”. At first, we used a barometer sensor and 3D optical-flow to estimate the user’s elevation. Both methods are relatively sensitive to elevation changes but both tend to drift. Moreover, 3D optical-flow methods are unable to detect vertical movement in an elevator. Therefore, we designed the following floor-change filter, which is based mainly on continuous changes in barometer readings (see Figure 5). For simplicity, we assume the barometer sampling rate is fixed; a common sampling rate of smartphones is 5–15 Hz. Initially, when no knowledge about the current floor if provided, the particles are spread among all floors. Algorithm 2 reports significant elevation changes in meter, while using adaptive filter for overcoming slow drifts in the barometer reading due to atmospheric changes.

**Algorithm 2:** Floor-change algorithm:
if the user had been going up or down, the algorithms estimate the elevation change between the current *z* and the last flat-floor parameter. As long as the user remains in a flat, minor changes in the measured elevation are omitted. A vertical velocity of [0.20,0.25] m per s, is a common value for $C_{up}$ and $C_{done}$ assuming a walking user.

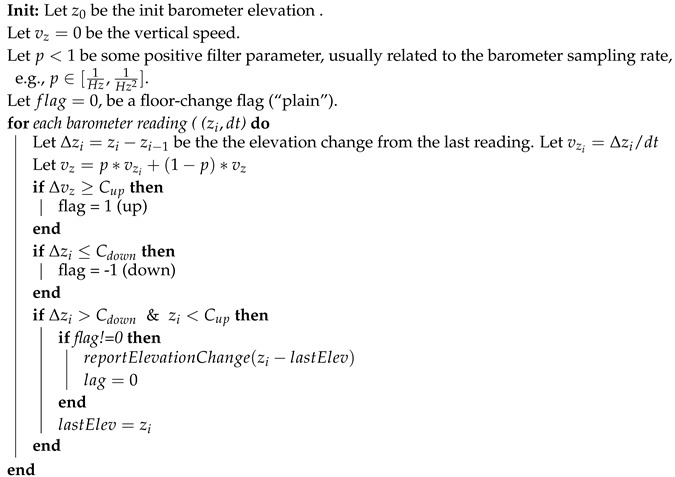



### 3.3. Velocity Estimation

**Figure 6:**
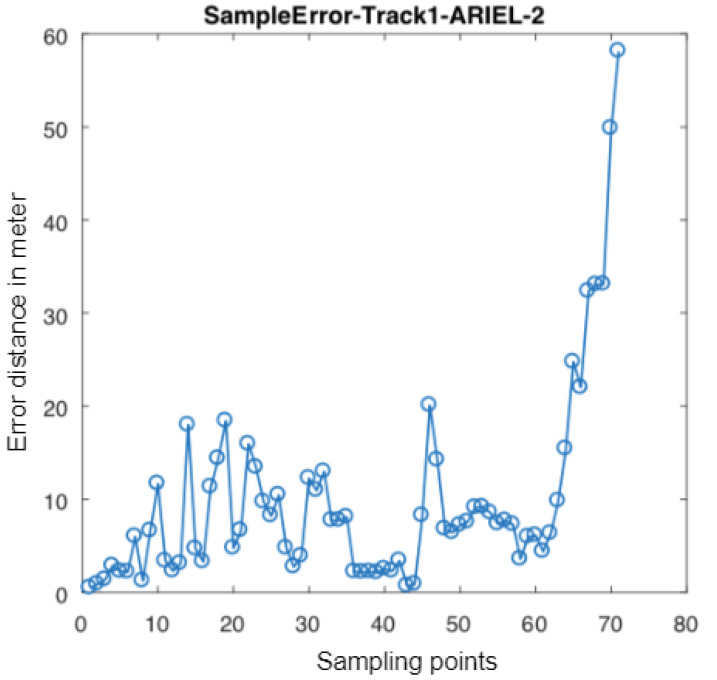
Error estimation of each of 70 points along the evaluation process. A travelator (moving walkway) in the evaluation path probably generated the large reported error from significant compass drift in the last few points.

Indoor navigation methods often use an inertial measurement unit sensor to implement a pedometer that detects the device’s global orientation and counts “steps.” However, such methods introduce significant inaccuracy in both the distance measured and the orientation (i.e., some steps are larger than others, and the device orientation only loosely correlates with the walking orientation). Therefore, we use optical flow with plains and range detection [29] to estimate the user movement with a high sampling rate. In regular indoor conditions with proper light and limited movement of the device—the optical-flow methods can allow a low drift of sub 2%. Recent tracking cameras such as Intel’s T265 (which uses stereo camera) reports an expected error of sub 1%. Yet, optical-flow based relative navigation systems are highly sensitive for major drifts. See [30] for a detailed accuracy evaluation of AR and VR platforms. The main causes of such drifts are: (a) Compass drift due to metal interference or local magnets (see Figure 6 for an example of a major error due to compass drift). (b) Visual orientation is also sensitive for drifts due to rapid movement or momentary blockage of the camera. (c) Poor light conditions. Figure 5 shows how sensitive to changes in the light conditions are such optical flow systems. (d) Robot-Kidnapped cases: in which a wrong loop-closer is performed which leads to major accuracy errors.

Although optical-flow is often insufficient for long-term visual navigation (e.g., Figure 5), in many short-term cases it tends to perform well. In other words: most of the major errors of velocity (as computed by optical-flow methods) are due to few cases of “major drifts”. Therefore, we suggest the following filter-algorithm which cope with the above drift sources and allows a robust and accurate velocity estimation using optical-flow methods.

In most cases Algorithm 3 simply reports the velocity computed by the optical flow method. Yet, in case of low confidence the algorithm suggests few options for the current velocity and device orientation. Having several optional velocities allows the particle filter to test them on the particles. In some cases this might require increasing the number of particles, yet such increment is momentary and the number of particles can be reduced back—as elaborated in the subsections below. The exact implementation of the confidence estimation (of the optical velocity) might be platform dependent. In our case we use the following parameters: (i) light condition. (ii) correlation between the gyro reported angular change and the optical orientation. (iii) a velocity rate which should be relatively close to the last velocity and within a walking (or running) rate.

**Algorithm 3:** Optical Velocity Algorithm: in cases of low confidence it will report several options of velocity and device orientation.

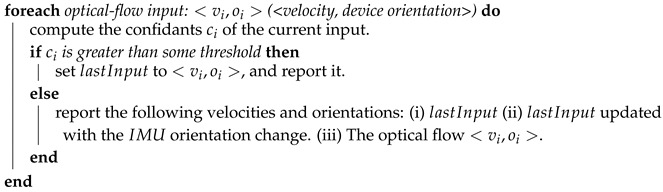



The optical flow was implemented using AR library. The STEPS implementation is using ARCore tool which allows: optical flow, plan detection and light estimation. We use the ARCore self motion tracking as the input source for velocity Algorithm 3. Combined, the particle filter can now take advantage of tracking capabilities such as “loop-closer” without the risk of having a wrong adjustment or a “Kidnapped Robot”, as presented in the subsections below.

### 3.4. Improved Sense Function

**Figure 7:**
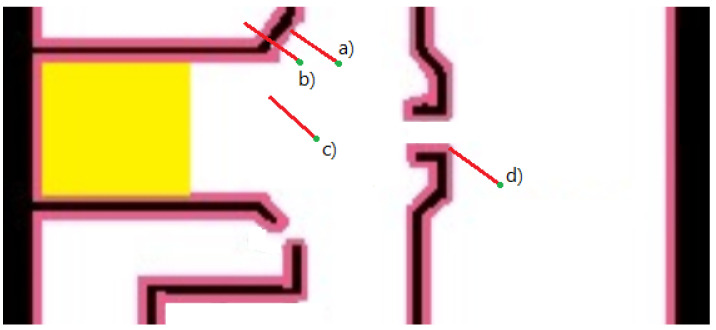
Consider the use of a single point light detection and ranging (lidar) sensor (or any other ranging sensor): The red vector represents such a range measurement (length and angle). Particles (**a**) and (**d**) represent good sensing points because they are within the right range (pink region). The grades of Particles (**b**) and (**c**) will be decreased. The case of Point (**b**) might be due to temporal blockage (e.g., human), whereas Point (**c**) most probably is in the wrong location.

The improved algorithm performs additional sensing of the range between the device’s camera and the closest obstacle (in the camera’s central orientation). Consider Figure 7, assuming the range between the device (in the camera direction) and the first obstacle (marked in a red vector) is known. We can use this vector to decrease the region of possible particles significantly. The sense measures the real distance of the device location to the nearest obstacles, such as walls or doors. The sense function examines, for each particle, whether a vertical obstacle exists on the map, and in the same distance and orientation from the particle location and regrades them respectively. Such sensing can be done using the camera’s auto-focus laser sensor (common in smartphones) or an AR measurement tool, specifically ARCore (or ARKit) due to their key feature, environmental understanding. This feature detects vertical surfaces and measures the distance to them. As expected, the measurement is noisy. Therefore, we added a fifth color to those described in the map constrains (Section 3.1) to add some error interval for the ranging noisy sensor. As shown in Figure 7, a pink interval is added to the walls (and other obstacles) on the map. Using the extra range-sensing data may reduce the area of possible particles significantly: from the white region to the pink region, allowing faster convergence of the particle filter algorithm and more accurate localization results overall.

### 3.5. Improving Compass Accuracy

The orientations that smartphones report often suffer from significant errors due to magnetic interference. To reduce the orientation inaccuracy related to compass noise and bias, the particle state may include an additional dimension to estimate the original compass bias and current drift. Initially, each particle starts with some Gaussian random value of compass bias. During the resampling process, each new particle is assigned a compass-related state according to the values of its nearest neighbors, with some minor noise. The particle then uses the smartphone’s compass-measured data, combined with its bias and drift, for the move function.

### 3.6. Sparse Sensing

Particle filter algorithms [31] rely on the assumption of a continuous flow of incoming data from the sensors. In fact, that sampling process cannot be achieved adequately because many localization problems are comprised of sparse sensing scenarios (i.e., scenarios in which data from the sensor is obsolete). In the context of this paper, assuming a localization algorithm is vision based, blocking the camera could cause serious ramifications because sampling works well only given the correct importance of the grades. The grades, of course, are obtained from the real world via the sensors. When such a scenario is detected, the particle filter is affected and reacts by adding random noisy movement to each particle according to the previously measured movement pace. The result of such a reaction will be more scattered particles that will cover for the momentary uncertainty.

### 3.7. Adjustable Particle Set

In the particle filter algorithm, increasing the number of particles allows improved representation of the probabilistic space, which leads to improved accuracy. On the other hand, the algorithm complexity correlates with the number of particles. Thus, we suggest an adjustable particle filter that adjusts the number of particles to the expected probabilistic space. In cases where there is a large region of possible solutions (e.g., init-stage in few floors), a large set of particles will be used. However, later—when the particle filter tends to converge—the number is reduced significantly, allowing a better practical run time with lower memory usage and lower average energy consumption.

### 3.8. Kidnapped Robot

Kidnapped Robot is a well known problem [32] that, in our system’s context, refers to a situation when the algorithm completely loses track of the real-world location and, thus, the evaluation function performs badly. To tackle this problem, we used a geolocation service (e.g., Google Maps geolocation API), which provides a method with “building level” accuracy, and used it as an anchor to the truth. In case our system reports an extremely different location from the one the used geolocation service reports, we reboot the system based on the geolocation service report, respective of the service’s expected accuracy. This feature proved useful in the IPIN-2019 competition (Section 4.4).

### 3.9. Indoor and Outdoor Classification

**Figure 8:**
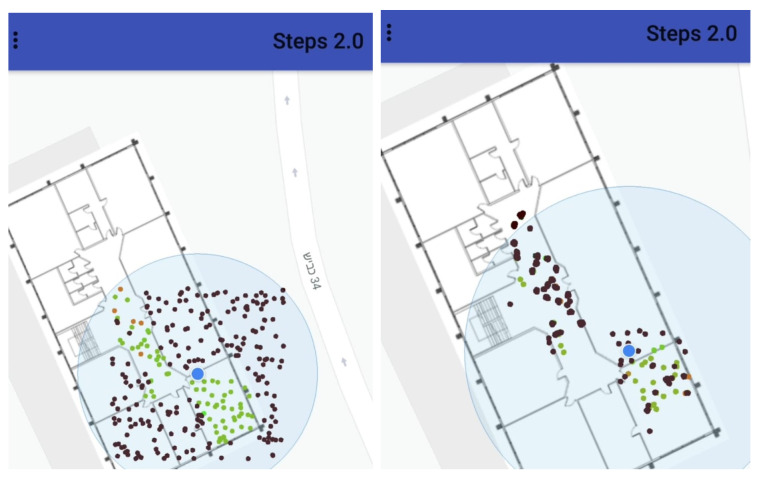
(**Left**): Particle initialization without indoor/outdoor sensing. (**Right**): The ability to classify between indoor and outdoor allows the algorithm to decrease the overall area of possible solution dramatically, leading to faster convergence with better accuracy.

There are several methods to detect if the user’s phone is indoors or outdoors. Using the GNSS maximal signal strength is one way to estimate if the phone has line of sight to the navigation satellite (see [33] for more details). Another method might be based on the phone’s light sensor because in daylight, outdoor light is usually stronger than indoor light (even on cloudy days), whereas at night, the opposite happens. By using such a method, we can add an increased sense evaluation, as presented in Figure 8.

### 3.10. Parameter Optimization

One of the most significant performance issues with the particle filter is the ability to optimize the algorithm’s parameters. The algorithm has a large number of parameters, among them the number of particles, system noises, regrade process intensity, and thresholds. Finding the optimum values for those parameters is a desired goal. Commonly, parameters are set manually, according to experiments and discretion. Instead, to allow an automatic process of parameter fine-tuning, we designed a complicated heuristic method based on a genetic algorithm. A genetic algorithm is a common method for optimization and search problems inspired by Darwin’s theory of evolution by natural selection [34]. A genetic algorithm simulates the natural selection process in a given population by three main steps:

- **Selection**: Select the fittest individual through the evaluation process.
- **Crossover**: Pair two selected individuals to populate the next generation. Their offspring will carry a genetic cargo that is a combination of their parents’ genes.
- **Mutation**: Create a mutation in the offspring’s genes from the previous step.

These three steps are repeated until the genetic algorithm simulation is completed.

In our genetic algorithm, each whole particle filter simulation represents an individual in the population, with the algorithm’s parameters set as the genes. The evaluation of each individual was determined by comparing its algorithm performance to a ground truth (ground truth; further discussed in Section 4.1). Therefore, individuals with a well-performing algorithm (i.e., those with more suitable parameters) will be selected for the crossover stage, and their fit genes will be preserved and improved in the next generation.

In more detail, first, each individual’s genes consist mainly of numerical values but also of whole functions as genes. For example, one gene holds the method for the best selection in the particle filter. However, that method will not necessarily be the same for two given individuals. In this way, we can test the preference of some methods over others, regarding evaluation in the selection process, the greatest concern is for the particle filter’s accuracy. However, we also need to consider its run-time performance, especially given that we present a real-time algorithm for mobile devices. Originally, the genetic algorithm was designed to run on a simulation platform. Recently, we found that by using the crowd-sensing concept, users may perform a distributed parameter optimization algorithm. For each site, users perform a navigation and evaluate the few variant sets of parameters (as computed by the simulated genetic algorithm). Evaluation without a ground truth can be done by testing the particles’ convergence rate. This way, each user can contribute to finding the optimal parameters for each specific site.

## 4. Experimental Results

To test the suggested localization framework, we performed a set of simulation and field experiments. By using three navigation competitions in which the suggested STEPS framework took part in 2018 and 2019, we allowed for reliable and comparable results.

### 4.1. Simulation

Having described the particle filter in the previous section, we now evaluate its performance. In the absence of available and accurate ground truth tools, most of the evaluation was done through a dedicated simulator. In this section, we present the details of our evaluation, evaluation methodology, and simulation results.

As previously described, the particle filter algorithm has several parameters. One purpose of the simulator is to enable optimization of those parameters by comparing different assignment options. In addition, we examine the algorithm’s performance, including the parameters of convergence speed, dealing with errors (noisy sensors, kidnapped robot, etc.), and typical accuracy. For this, the simulator allows loading raw data of walking recordings. Thus, it simulates the particle filter algorithm performance on this data, together with an analysis of the algorithm performance and visual display.

**Figure 9:**
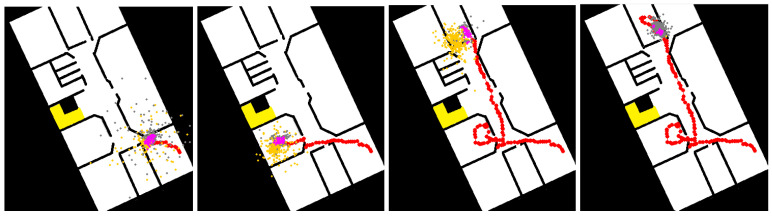
Particle filter algorithm progress over time (from left to right). The optical sensor data affected the orange particles, whereas the smartphone’s internal compass affected the gray particles. Having both particle types allows a more robust solution.

The simulation also was handy when we wanted to examine not only the particle filter and its parameters, but also other factors, such as the influence of the sensor we used on the overall results. For example, we wanted to test which orientation sensor we should use to calculate the user movement path—the smartphone’s built-in compass or its already-calculated position from the optical flow virtual sensor. Figure 9 shows the simulator state over time, illustrating these two options and their reliability over time.

**Figure 10:**
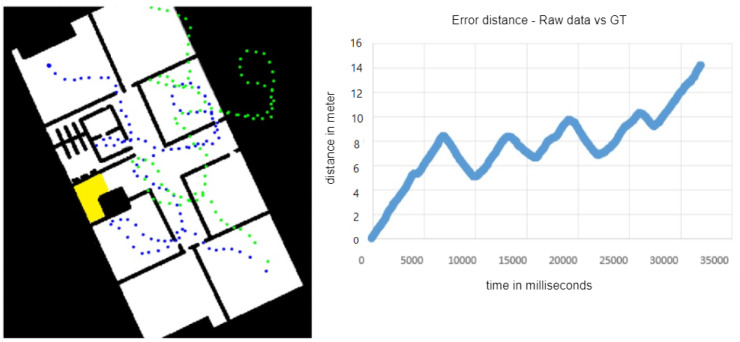
(**Left**): The green dots are the raw data, and the blue dots are the ground truth. (**Right**): The graph shows the error distance between them.

**Figure 11:**
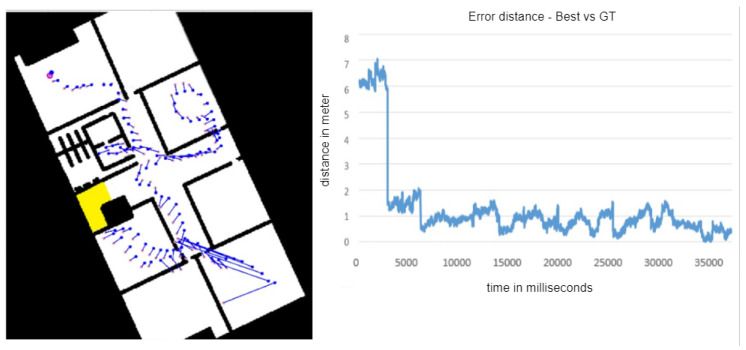
(**Left**): The pink dots are the STEPS reported locations, and the blue dots are the ground truth. (**Right**): The graph shows the error distance between them, as well as the length of the lines between each coupling of a ground truth point and its related STEPS point.

Figure 8, Figure 9, Figure 10 and Figure 11 represents a 20 × 40 m University building in which lab experiments and simulations were performed. Eventually, the method we embraced was the smartphone’s built-in compass, mainly due to the high sensitivity of the optical flow’s virtual sensor, which different lighting and fast movements easily affected and thus could lead to serious faults.

A core feature of the simulation is its ability to generate fake raw data. An “eyed” measurement is not enough to properly test the algorithm’s performance; the need for a ground truth measurement comparison is well understood. The simulator is capable of generating raw noisy data, as shown in Figure 10, as well as real data alongside the ground truth of that data. This enabled us to test our algorithm accuracy more precisely by comparing its reports to the ground truth. As demonstrated in Figure 11, our algorithm lowered the maximum error from approximately 16 m to approximately 7 m, but only for a short time, after which the report stabilized with a maximum error of approximately 1.5 m. All the presented simulations figures are of experiments that was conducted in a building with an area of 800 square meter.

### 4.2. Case Study: Microsoft Indoor Localization Competition

**Figure 12:**
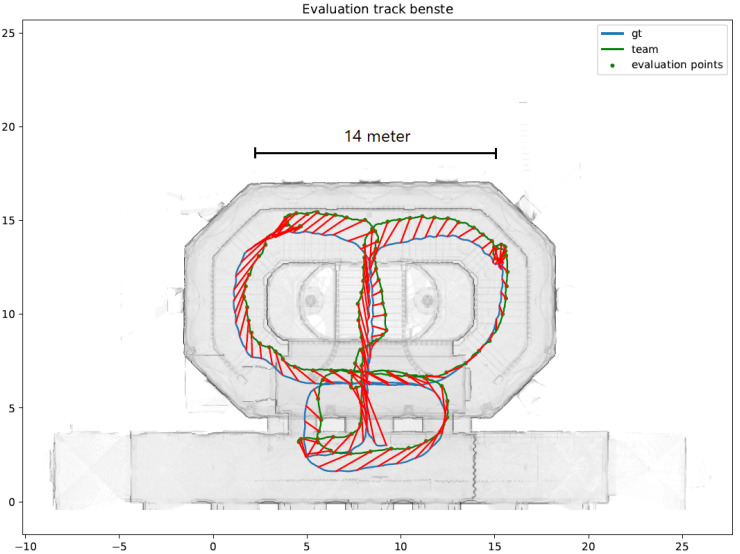
The STEPS path with the 2D error with respect to a centimeter-level lidar-based ground truth.

**Figure 13:**
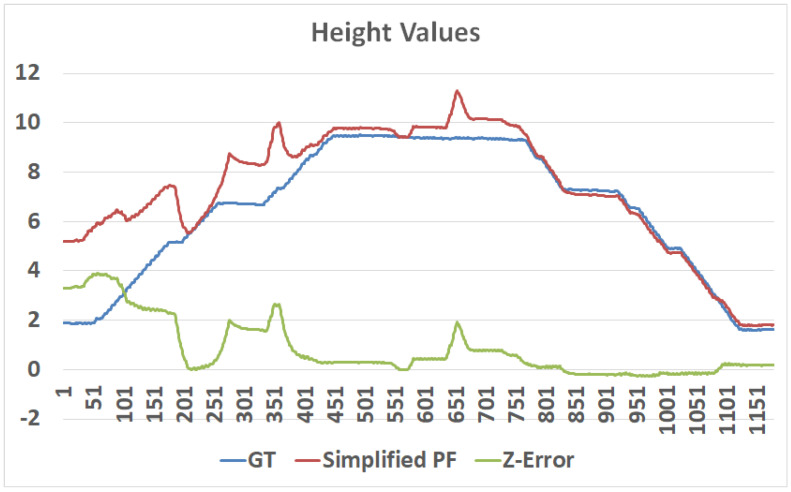
A 3D particle filter in action: an (about) 120 s test, in which the position converged from an error of 3.5 m to a submeter within 40 s and to (about) 0.1 m error after 80 s. Note: the z value is defined according to the best particle height (no filter) in order to demonstrate the convergence process.

**Figure 14:**
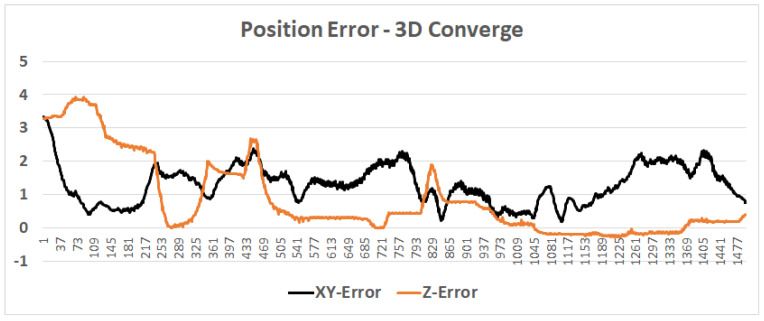
Particle filter 3D convergence: an (about) 120 s test, in which the position converged from a height error of 4 m to a sub-meter; upon having the proper height, the horizontal position converged, as well.

**Figure 15:**
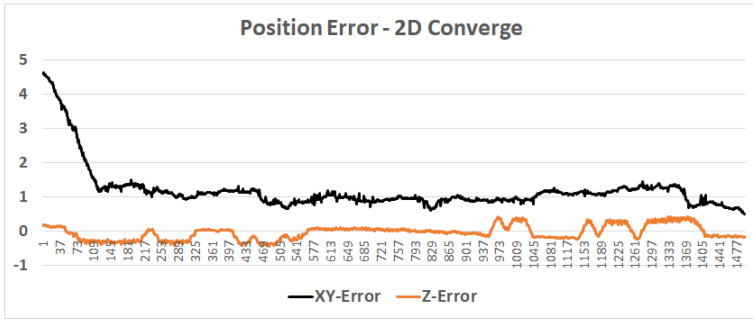
Particle filter 2D convergence: Assuming the correct floor is known, the horizontal position convergence moves from an error of 4.5 m to about 1.3 m within 10 s (about 15 steps). During the rest of the test, the horizontal error is about 1 m, whereas the vertical error averages less than 0.5 m.

**Figure 16:**
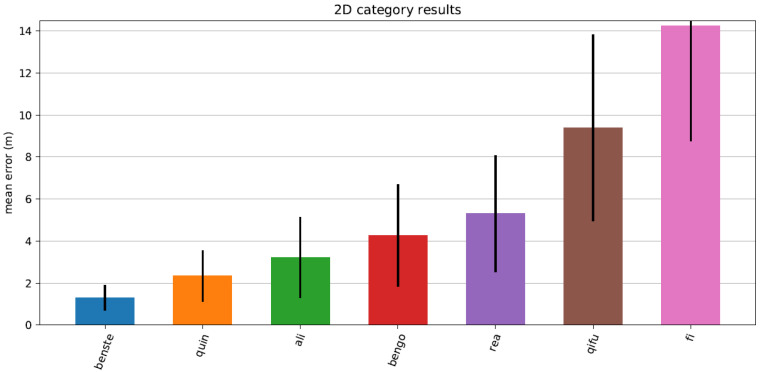
2D error in meters as tested in Microsoft Indoor Positioning competition 2018. The STEPS (benste) reached an average accuracy of 1.3 m.

**Figure 17:**
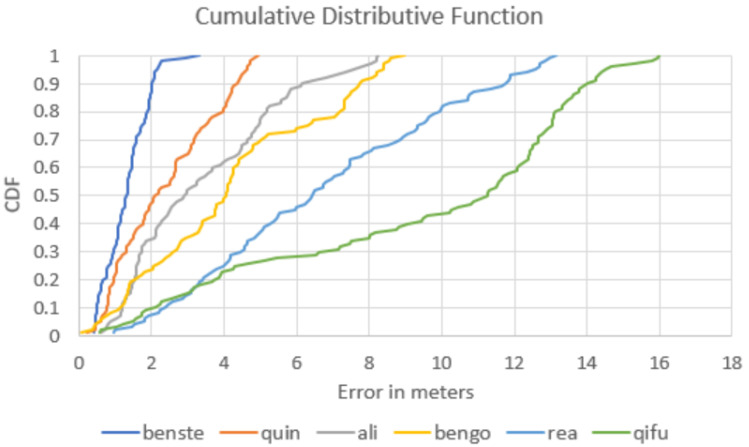
Cumulative Distributive Function: of the 2D error in meters as tested in Microsoft Indoor Positioning competition 2018. The STEPS (benste) reached an average accuracy of 1.3 m.

Since 2014, Microsoft Corporation has organized an annual Indoor Localization Competition (see [6] for the 2014–2017 evaluations). In the 2018 competition, we implemented a preliminary version of the suggested particle filter (STEPS). In general, the system was design to improve existing IPS (such as Google’s Indoor Maps API) from an expected accuracy of 10 m to 20 m to an accuracy for 1 m to 2 m (3D). Overall, the system performed as expected, allowing a rapid convergence—within 10 to 15 s (for 15–20 steps)—of the particle filter. Figure 12 presents the 2D evaluation of the system with respect to the ground truth, and Figure 13 presents the z-convergence process, in which the floor position was accurately found after about 40 s. Figure 14 and Figure 15 show the different convergence natures of the particle filter in the 3D case (when the floor is unknown) and the 2D case (when the floor is given).The evaluation of our algorithm led us to the first place in the competition, see Figure 16 and Figure 17. STEPS system result marked as benste. A list of the other competitors and their system techniques is presented below:

- **QuIn:** The proposed system uses mobile’s inertial sensors to track user location with data fusion algorithm to fuse inertial data.
- **QiFu:** The proposed system exploits WiFi and geomagnetic fingerprinting along side with IMU and computer vision techniques.
- **Rea:** The proposed system uses time-of-flight Wireless Indoor Navigation System, that estimates the position of commercial off-the-shelf devices such as smartphones, tablets and laptops using pure commercial off-the-shelf WiFi Access Points in near real-time.
- **Fi:** The proposed system based on human stride-model analysis combining with geomagnetic positioning and WiFi fingerprinting technology.
- **Ali:** The proposed system is an infrastructure free technique which exploits the location and tracking information of WiFi and PDR with sparse geomagnetic tagging of the environment.
- **BenGo:** The proposed system uses particle filter which combine RF fingerprinting, odometry, visual landmarks and map constraints.

The competition was conducted in a building with an area of about 1000 square meter.

### 4.3. Case Study: 2018 International Conference on Indoor Positioning and Indoor Navigation (IPIN)

**Figure 18:**
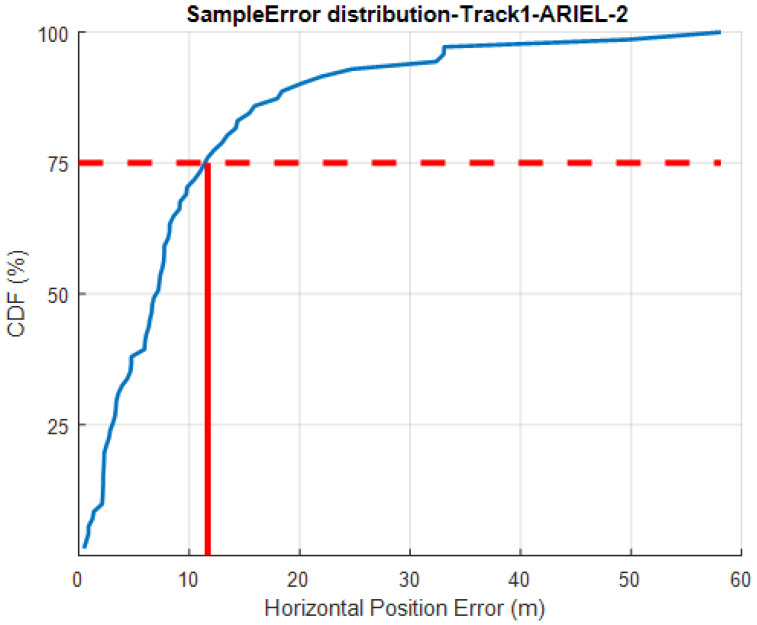
STEPS system sample error distribution, measured at the Microsoft Indoor Localization Competition.

In September 2018, an indoor positioning competition was held in a large shopping mall at Nantes, France as part of the 2018 IPIN Conference. The on-site competition had two tracks (with and without a camera). Naturally, we took part in the camera-based positioning track, implementing a preliminary version of the algorithm on a Tango-based Android phone (the initial starting position given to competitors). The evaluation was conducted over about 70 known waypoints (each with a known 3D global position) and a path on three floors (more than 1 km long). The particle filter localization algorithm was able to maintain a relative (4 m to 12 m) accuracy averaging 7.2 m (see Figure 6 and Figure 18). The overall evaluation of our algorithm led us to first place in the competition. Notably, the GoIn algorithm [35] won s place with almost similar results.

### 4.4. Case Study: 2019 IPIN

**Figure 19:**
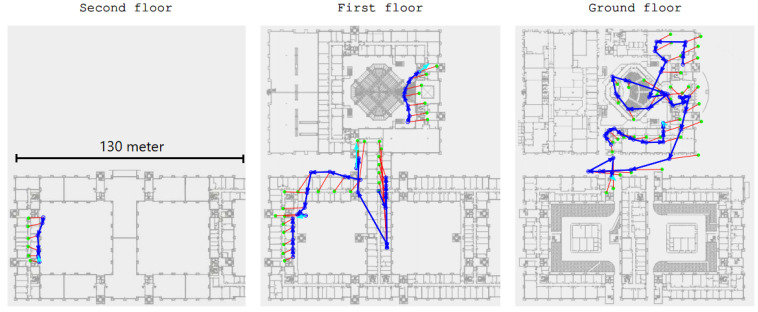
(**Left**): s floor. (**Center**): First floor. (**Right**): Ground floor. Green dots are the ground truth, blue dots are the STEPS system report, and red lines are the report’s error distance.

In September 2019, the IPIN indoor positioning competition was held in a large, public indoor multi-floor building at Pisa, Italy. We took part in the smartphone-based track, implementing a version of the STEPS algorithm on an Android phone. Competitors were provided with the initial starting position and a detailed area map. The evaluation was conducted over about 70 known waypoints (each with a known 3D global position) and the path comprised three floors. The particle filter localization algorithm was able to maintain a relative 11 m accuracy on average (Figure 19). The overall evaluation of our algorithm led us to s place in the competition.

**Figure 20:**
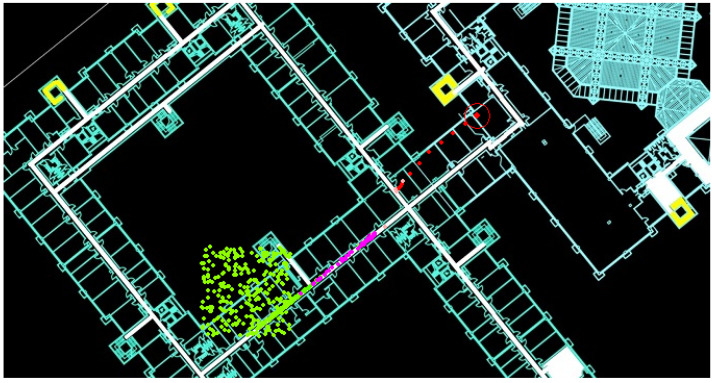
Negative effect of using geolocation service: The **red double circle** marks the true location while all the particles (marked as **green dots**) are located far away due to low accuracy geolocation service signals.

**Figure 21:**
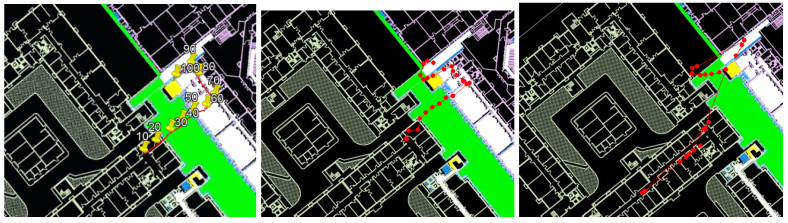
The negative effect of using geolocation service. (**Left**): ground truth. (**Center**): STEPS report without geolocation service resample. (**Right**): STEPS report with geolocation service resample. In this example, a significant positioning error in the geolocation service caused a major drift in the system because the true position was often outside of the particle filter ROI.

The main findings regarding the STEPS’s relatively low accuracy in the 2019 IPIN competition were due to some reliance of the system on geolocation service signals. As previously explained, our system relies in a certain way on software sensor. This sensor reports the current coordinates with some accuracy; its purpose is to avoid cases of Kidnapped Robot. The problem in this competition trial was that the algorithm did not consider the service’s reported accuracy. In action, the location API provided reports with very low accuracy, which caused severe drift in the STEPS system (Figure 20 and Figure 21). Thus, the question is not whether, but only to what extent, to rely on the system.

**Figure 22:**
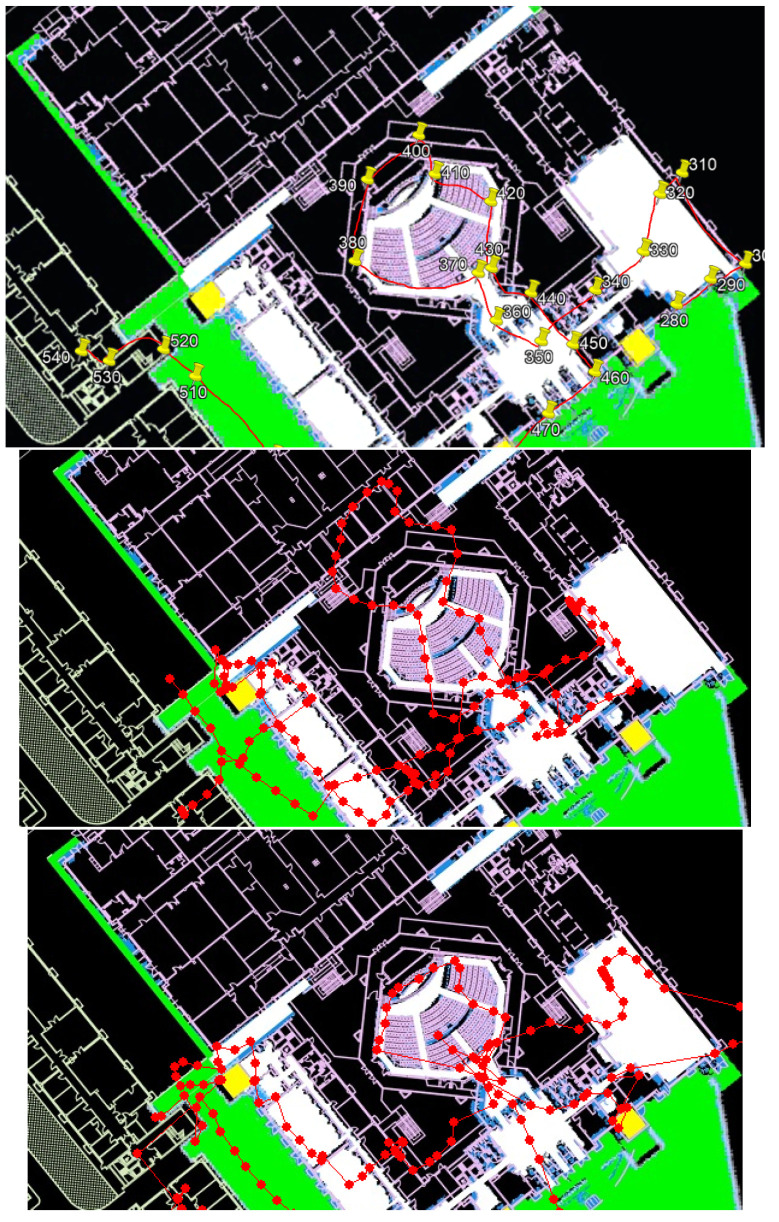
The benefit of using a global (sparse) position system as a geolocation service for the resample process. (**Top**): ground truth. (**Center**): STEPS report without geolocation service resample. (**Bottom**): STEPS report with geolocation service resample. In this example, using geolocation resampling helped the system overcome significant drifts.

This is not all bad news. It is important to mention the quality of relying on the location API sensor. As shown in Figure 22, thanks to the sensor, the STEPS managed to avoid the Kidnapped Robot scenario.

### 4.5. Energy Consumption and Privacy Evaluation

In this subsection we cover the overall performance of the suggested STEPS framework in terms of energy consummation and privacy. Based on field experiments, the STEPS implementation consumes approximately 12% battery per hour, as running on Samsung Galaxy S8 with a screen “always on display” in low brightness state. We conclude that the energy consumption of the STEPS application resembles GNSS navigation applications and can allow a massive indoor navigation use of 2–3 h without draining the smartphone’s battery too much.

The suggested framework has a high frame rate positioning of [10–30] Hz and can work in fully offline mode (in which no communication is being transmitted from the smartphone). In this mode the user’s privacy is not compromised.

## 5. Discussion

The challenge of finding an accurate, robust, and cost-effective indoor positioning solution is highly motivated by a wide range of applications from LBS. In this paper, we presented an optical-based navigation system that seems to solve most of the algorithmic problems and present several cases in which we tested the system. The overall performance of the presented indoor navigation framework is reaching the accuracy level of an outdoor commercial GNSS solution. Naturally, there remains a massive amount of delicate engineering needed to convert the suggested framework to a commercial navigation system. However, with the ever-improving sensors and virtual sensors in our mobile phones, and the increase in their computing power, it seems that, in a few years, a global indoor navigation solution will become part of our mobile phone sensors— just like edge-based deep learning capabilities evolved from an academic framework to a widely used commercial solution.

## Figures and Tables

**Table 1:** Sensors for indoor positioning which are common in smartphones. The data was taken from several popular smartphones such as Samsung’s Galaxy S7, S8 and S9.

Sensor List			
**Sensor Type**	**Main Use for Positioning**	**Sampling Rate**	**Expected Error**
Magnetometer (compass)	Orientation: Yaw - E-compass	10 Hz	[2–10] degrees
Magnetometer (strength)	Magnetic Field	10 Hz	NA
IMU	Orientation: Pitch and Roll	50 Hz	[1–2] degrees
Barometer	Relative Elevation	10 Hz	[0.3–1.0] m (relative)
Camera	Optical flow	30 Hz	[1–3]% (relative)
Range	Distance from wall or floor	10 Hz	2% upto 4 m
GNSS	Indoor Global Position	1 Hz	[5–50] m in indoors
BLE	RSSI Fingerprinting	1 Hz	[3–10] m
WiFi	RSSI Fingerprinting	[0.3–1] Hz	[3–15] m
4G	RSSI Fingerprinting	1 Hz	[10–50] m
